# Pavement Overrides the Effects of Tree Species on Soil Bacterial Communities

**DOI:** 10.3390/ijerph18042168

**Published:** 2021-02-23

**Authors:** Yinhong Hu, Weiwei Yu, Bowen Cui, Yuanyuan Chen, Hua Zheng, Xiaoke Wang

**Affiliations:** 1 State Key Laboratory of Urban and Regional Ecology, Research Center for Eco-Environmental Sciences, Chinese Academy of Sciences, Beijing 100085, China; huyinhong14@mails.ucas.ac.cn (Y.H.); 18514088911@163.com (W.Y.); bwcui_st@rcees.ac.cn (B.C.); zhenghua@rcees.ac.cn (H.Z.); 2 University of Chinese Academy of Sciences, Beijing 100049, China; 3 CAS Key Laboratory of Aquatic Botany and Watershed Ecology, Wuhan Botanical Garden, Chinese Academy of Sciences, Wuhan 430074, China; chenyuanyuan@wbgcas.cn

**Keywords:** impervious pavement, 16S rRNA, bacterial diversity, pervious pavement, tree species

## Abstract

Human disturbance and vegetation are known to affect soil microorganisms. However, the interacting effects of pavement and plant species on soil bacterial communities have received far less attention. In this study, we collected soil samples from pine (*Pinus tabuliformis Carr.*), ash (*Fraxinus chinensis*), and maple (*Acer truncatum Bunge*) stands that grew in impervious, pervious, and no pavement blocks to investigate the way pavement, tree species, and their interaction influence soil bacterial communities by modifying soil physicochemical properties. Soil bacterial community composition and diversity were evaluated by bacterial 16S amplicon sequencing. The results demonstrated that soil bacterial community composition and diversity did differ significantly across pavements, but not with tree species. The difference in soil bacterial community composition across pavements was greater in pine stands than ash and maple stands. Soil bacterial diversity and richness indices decreased beneath impervious pavement in pine stands, and only bacterial richness indices decreased markedly in ash stands, but neither showed a significant difference across pavements in maple stands. In addition, bacterial diversity did not differ dramatically between pervious pavement and no pavement soil. Taken together, these results suggest that pavement overwhelmed the effects of tree species on soil bacterial communities, and had a greater effect on soil bacterial communities in pine stands, followed by ash and maple stands. This study highlights the importance of anthropogenic disturbance, such as pavement, which affects soil microbial communities.

## 1. Introduction

Of the world’s population, 55% lived in urban areas in 2018, and this is projected to reach 68% by 2050 [1]. Impervious pavement covered by roads and buildings continues to increase because of population urbanization [2]. It has been estimated that artificial impervious areas reached nearly 800 thousand km^2^ globally by 2018, more than 2.5 times that in 1990 [3]. Impervious surfaces have adverse effects on urban ecosystems, such as urban waterlogging and the heat island effect [4]. Although green spaces [5] and pervious pavement [6] are used widely to mitigate the adverse influences of impervious surfaces, biodiversity aboveground, including that of plants and birds, has declined with the increase in impervious surface areas in cities [7,8]. However, the effects of impervious surfaces on biodiversity underground, such as soil microbial diversity, are unclear.

Soil microorganisms play a key role in mediating the global cycling of nutrients and maintaining ecosystem services [9]. However, impervious pavements affect soil negatively, including depleting soil total carbon and nitrogen and increasing its salinization [10]. Further, the installation of pavement decreases soil microbial biomass by changing temperature, moisture, and nutrients underground [11]. Soil microbial enzymatic activities also are restricted under impervious pavements [12]. Moreover, our previous study about the influences of land cover types on soil microbial communities found that bacterial diversity decreased under impervious surfaces, and bacterial community structure was altered compared to that in lawns and areas with shrub coverage in cities [13]. However, the areas of these studies were road or residential quarters where topsoil was removed, and there have been no studies on soil bacterial community composition and diversity beneath pavements based on long-term field experiments.

The diversity and community activities of microorganisms that live belowground contribute significantly to shaping plant biodiversity and function aboveground [14]. In turn, plant species are a critical factor in determining soil microbial biomass and function diversity [15]. Each tree species is colonized by specific bacterial and fungal populations, which root exudates determine [16]. Human interference, such as the installment of impervious pavement, has effects on creatures both above- and belowground [12,17]. The presence of impervious pavement has been found to reduce deciduous tree species’ growth, inhibit plant photosynthesis, and accelerate leaf budburst by altering micro-environmental conditions (e.g., soil nutrients, temperature, and aeration) [18]. However, the intensity of pavement’s effects on plants’ physiological and growth characteristics has been demonstrated to vary with tree species [17,19]. Similarly, the Biolog Ecoplate and phospholipid fatty acid profiles methods showed that both pavement and tree species determined soil microbial community structure and functional diversity [20]. Further, changes in soil fungi: bacteria ratio, microbial carbohydrates, and amino acids beneath pavements differed between pine and ash [20]. However, it remains uncertain how the interaction effects of pavement and tree species influence soil bacterial communities.

Pervious pavements are used widely in cities to mitigate urban waterlogging and the heat island effect [21,22], and have different influences on plants aboveground and soil microorganisms below [23]. Although plant growth decreased beneath pervious pavement to the same extent as impervious pavement because of the increased temperature [18], it favors microbial communities compared to impervious pavements [13]. With respect to microbial community structure and functional diversity, pervious pavements can also mitigate the adverse effects of impervious pavements on the soil in both coniferous and deciduous stands [20]. 

In this study, a field experiment was performed in suburban Beijing, China to assess the way pavement, tree species, and their interactions influence soil bacterial communities. The soil bacterial 16S rRNA gene (V3-V4 regions) was sequenced to assess soil bacterial diversity and community structure. We monitored soil and surface temperature continuously, and used soil properties to explore their influence in bacterial communities. We hypothesized that: (1) Pavement reduces soil bacterial diversity and alters bacterial community composition; (2) soil bacterial community composition and diversity vary with tree species, and (3) pervious pavement can reduce the adverse effects of impervious surfaces on microbial communities.

## 2. Materials and Methods

### 2.1. Study Area and Experimental Design

A five-year field study was performed in Zhangtou village, Changping District, which is located in suburban Beijing (40°12′ N, 116°08′ E). The mean annual precipitation in the region is 542 mm and the annual mean air temperature is 12.1 °C. The experimental site had an area of 3510 m^2^ that consisted of agricultural lands planted with wheat and maize for many years before the experiment. Soils at the study sites were classified as sandy loam. The initial experimental soil characteristics, including soil pH, bulk density, organic matter, and available phosphorus and potassium, were described in our previous study [18]. 

In April 2012, the farmland selected was divided into three equal areas and covered with different bricks: One with no pavement (i.e., control), one paved with pervious bricks, and one paved with impervious bricks of the same size (Figure S1). Both bricks’ length, width, and height were 20 cm, 10 cm, and 6 cm, respectively. The bricks were made of clay, sand, and coal ash and were produced by Beijing Yataiyuhong Technology Development Co., Ltd. The pervious brick (permeability > 0.4 mm s^−1^) is coarse and porous on the surface, but the permeability of impervious brick is nearly zero.

Three tree species, pine (*Pinus tabuliformis Carr.*), ash (*Fraxinus chinensis*), and maple (*Acer truncatum Bunge*), were chosen to plant in each block because (1) all of them are popular in urban areas of northern China and (2) they represent two distinct species: Pine is coniferous, while ash and maple are deciduous. Each block was divided further into three equal plots. Pine, ash, and maple seedlings with similar basal diameter and height that were cultivated for one year were selected to plant in the study area. In each plot, 18 seedlings of each species were planted in 4 parallel dislocation rows in an east–west direction with a density of 2 × 2 m (Figure S1). Pits (0.2 × 0.2 m) were dug on the corresponding plant point before the pervious and impervious bricks were covered tightly on the soil surface side by side. 

The soil temperature was measured continuously at 30 min intervals at a depth of 20 cm using a Campbell CR1000 data logger under impervious, pervious, and no pavements in the pine, ash, and maple stands, respectively.

### 2.2. Field Sampling and Soil Chemical Analysis

In October 2017, 5 years after the pavement was laid and the trees planted, three subplots (2 m × 2 m) as replicates were established in each plot. Five soil cores (0–20 cm) that were 20 cm from the tree trunks were taken from each subplot at random, and were mixed into a composite sample. In total, 27 composite samples were obtained. The composite samples were placed in sterile plastic bags and transported to the laboratory on ice, where the stones and roots were removed, after which the samples were passed through a 2 mm sieve. The sieved soil samples were divided into two subsamples. One was air-dried to determine the soil chemical properties, and another one was kept refrigerated at −80 °C before DNA extraction. 

Soil variables, including pH, total carbon (TC), total nitrogen (TN), available phosphorus (AP), available potassium (AK), NH_4_^+^-N, and NO_3_^−^-N, were determined in the laboratory. The methods used to determine the study area’s soil chemical properties have been described previously [24].

### 2.3. DNA Extraction, Illumina MiSeq Sequencing and Bioinformatics Analysis

The DNA of each sample was extracted from 0.5 g soil with the FastDNA® SPIN kit (MP Biomedicals, Santa Ana, CA, USA) following the instructions in the accompanying manual. The concentration and quality of the genomic DNA were checked using a Nanodrop ND-1000 spectrophotometer (NanoDrop Technologies, Wilmington, DE, USA). The DNA samples extracted were sequenced on an Illumina MiSeq sequencer (Illumina Inc., San Diego, CA, USA). The methods for PCR amplification and 16S rDNA sequencing are shown in S1.

The raw data were managed with an in-house pipeline (http://mem.rcees.ac.cn:8080/ (accessed on 20 February 2021)) equipped with a series of bioinformatics tools. The methods of sequence processing are described in detail in S1, and the raw sequence data were deposited in the NCBI Sequence Read Archive database (accession number: PRJNA684697).

### 2.4. Statistical Analysis

All statistical analyses were conducted using R version 3.6.0 (R Core Team, 2018). Two-way ANOVAs were performed using the “aov” function to test the effects of pavement, tree species, and their interactions on soil properties and bacterial diversity, followed by post hoc comparisons using Turkey’s HSD test. Non-metric multidimensional scaling (NMDS) ordinations based on the Bray–Curtis distances using the function “metaMDS” with 999 permutations were conducted to investigate the difference in soil bacterial community composition among pavements and tree species. To distinguish the contributions of pavement and tree species to the variations in bacterial community composition, variation partitioning analysis (VPA) was conducted using the “vegan” package in R. The relation between soil bacterial community composition and soil variables was assessed by performing a canonical correspondence analysis (CCA) (“cca” function) and Mantel test (“mantel” function) with the Bray–Curtis dissimilarity measurement. Analysis of similarity (ANOSIM) was performed further to test whether bacterial community composition differed significantly among pavements in blocks with different tree species with the “anosim” function. A heatmap was generated using the “pheatmap” function. A linear mixed-effects model was used to investigate the relations among soil properties and soil bacterial diversity and richness indices. Models with the random effect of “plot” and the fixed effects of soil temperature, pH, TC, TN, NH_4_^+^-N, NO_3_^−^-N, AP, and Shannon and Chao 1 indices, were performed using the “lmer” function.

## 3. Results

### 3.1. Soil Properties

**Figure 1:**
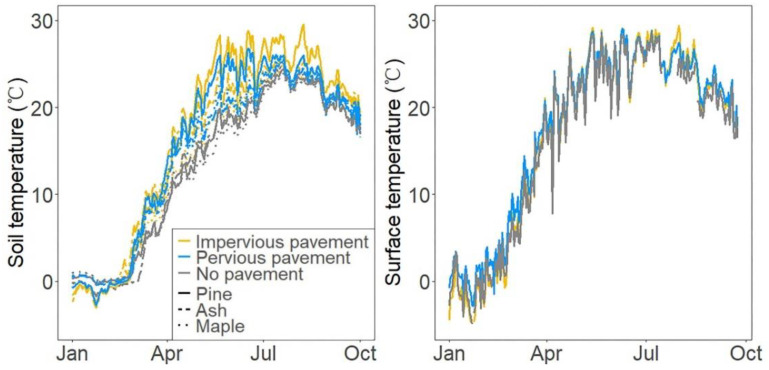
Daily mean soil temperature (°C) and surface temperature under different pavements in pine, ash and maple blocks from January to October 2017.

The soil and surface temperatures under impervious, pervious, and no pavement in ash, pine, and maple stands from January to October 2017 are shown in Figure 1. The soil temperature fluctuated with the season, as expected, and was correlated with surface temperature (Figure 1). There was no significant difference in surface temperature among pavements (Figure 1). However, the pavement had significant effects on soil temperature (Table S1), as it increased significantly beneath impervious and pervious pavement blocks compared to no pavement blocks, although it was similar between impervious and pervious pavement blocks. Moreover, tree species had no effects on soil temperature (Table S1).

Impervious pavement increased the content of soil NO_3_^−^-N, but decreased the concentration of AP and AK (Figure S2). Soil NO_3_^−^-N concentrations beneath impervious pavement were approximately twice as great as beneath no pavement, but AP showed the opposite trend. However, there were no statistical differences in soil pH, TC, TN, and NH_4_^+^-N among the different pavements (Figure S2). Moreover, tree species had no effect on any of the soil properties (Figure S2).

### 3.2. Bacterial Community Composition and Diversity

**Figure 2:**
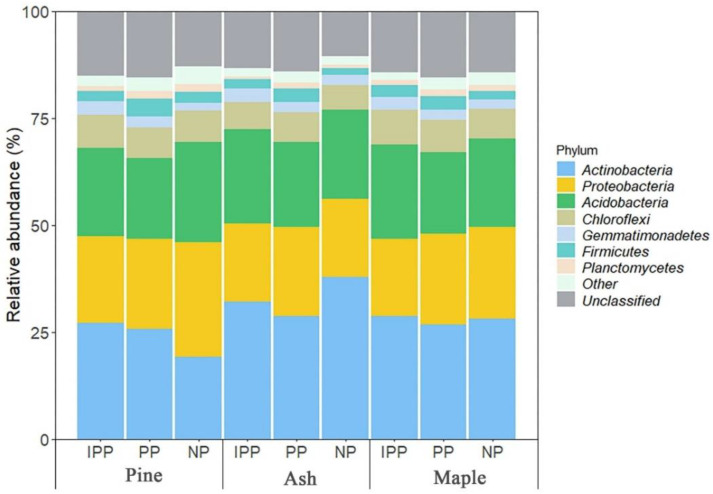
Soil bacterial community composition shown as relative abundance at phylum level. “Others” include phyla with less than 1% relative abundance.

A total of 394,092 high-quality V3-V4 region gene sequences were obtained after data filtering, which were clustered into 754 genera, 237 families, 99 orders, 75 classes, and 36 phyla. The dominant phyla across all samples were *Actinobacteria* (18.6–41.3%), *Proteobacteria* (11.6–27.5%), *Acidobacteria* (17.1–25.1%), *Chloroflexi* (5.0–9.7%), *Gemmatimonadetes* (1.2–4.5%), *Firmicutes* (1.3–5.4%), and *Planctomycetes* (0.3–2.3%), which accounted collectively for 79.0–88.6% of all taxon sequences (Figure 2). The heatmap analysis showed that soil bacterial communities of all samples were clustered into five groups at the genus level (Figure S3). The soil samples were clearly distinct among pavement types rather than tree species. *Gp16* and *Gp6* was the primary genus across all soil samples. *Gaiella* and *Gemmatimonas* were also found consistently in greater abundance in impervious and no pavement samples. Moreover, *Pseudomonas* was the dominant genus in the pervious pavement sites.

**Figure 3:**
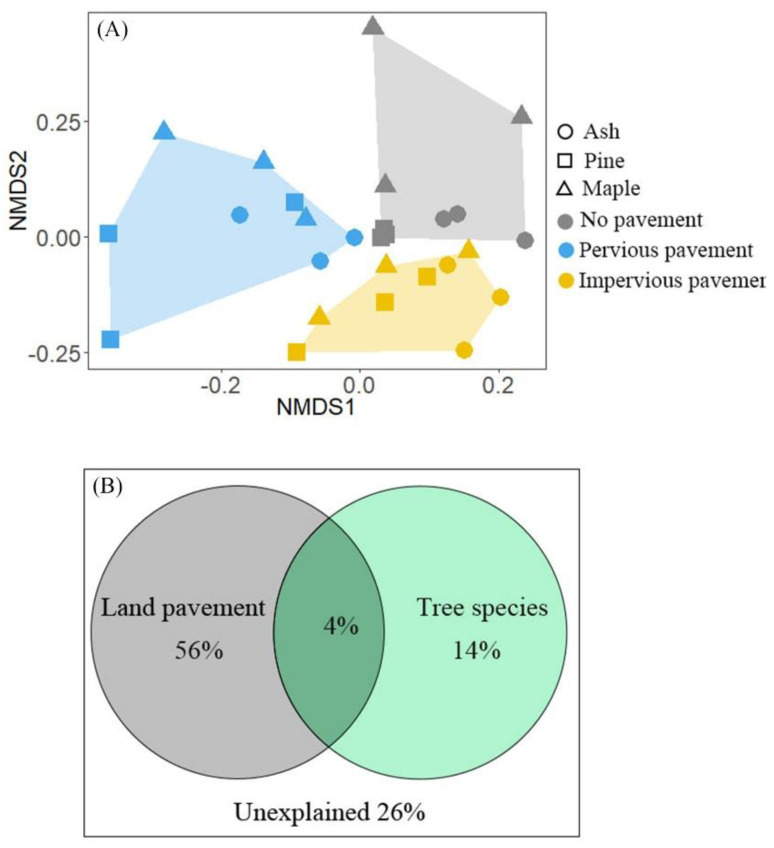
(**A**) Non-metric multidimensional scaling (NMDS) analysis based on the Bray-Curtis dissimilarity matrices showing the soil bacterial community composition sorted by pavement and tree species. (**B**) Variance partitioning analysis (VPA) results illustrate the proportion of variation explained in the bacterial community composition pavement, tree species, and the interactions between them.

Three distinct clusters were observed in the NMDS plots of the entire community, and were distributed in an orderly fashion from no pavement to impervious pavement samples (Figure 3A). However, samples from the same tree species were not grouped together. The results of VPA analysis revealed further that pavement alone explained 56% of the variation in soil bacterial community composition, while tree species explained only 14% of the variation (Figure 3B). In addition, pavement and tree species collectively explained 4% of the variations in bacterial community composition (Figure 3B). Pavement and tree species combined contributed a total of 74% of the variation in soil bacterial communities, leaving 26% unexplained (Figure 3B). The results of ANOSIM also revealed that soil bacterial communities differed among pavements (*R* = 0.511, *p* = 0.001), but were similar among tree species (*R* = 0.127, *p* = 0.081). Moreover, the differences in soil bacterial community composition among pavements in pine (ANOSIM: *R* = 0.605, *p* = 0.001) stands were greater than those in ash (ANOSIM: *R* = 0.514, *p* = 0.013) and maple (ANOSIM: *R* = 0.440, *p* = 0.019) stands, indicating that the soil bacterial communities in pine stands are more sensitive to pavement than those in ash and maple stands.

### 3.3. Soil Bacterial Diversity under Different Pavements

Soil bacterial diversity and richness were assessed using the Shannon index and Chao 1 index, respectively. The soil under impervious pavement in pine and maple blocks had the lowest bacterial Chao 1 richness (Table 1), and the Shannon diversity estimators were lowest in the soil beneath impervious pavement, as well (Table 1). The results of a two-way ANOVA revealed that pavement rather than tree species affected soil bacterial diversity (pavement: F = 10.006, *p* = 0.001; tree species: F = 2.031, *p* = 0.078) and richness (pavement: F = 7.018, *p* = 0.006; tree species: F = 0.031, *p* = 0.964). Interestingly, both the soil Shannon and Chao 1 indices differed significantly between impervious and no pavements in pine stands. However, only the soil Chao 1 index differed among pavements in ash stands (Table 1). Conversely, the soil Shannon diversity and Chao 1 indices did not differ significantly among pavements in maple stands (Table 1). 

### 3.4. Relations between Soil Characters and Bacterial Communities

**Figure 4:**
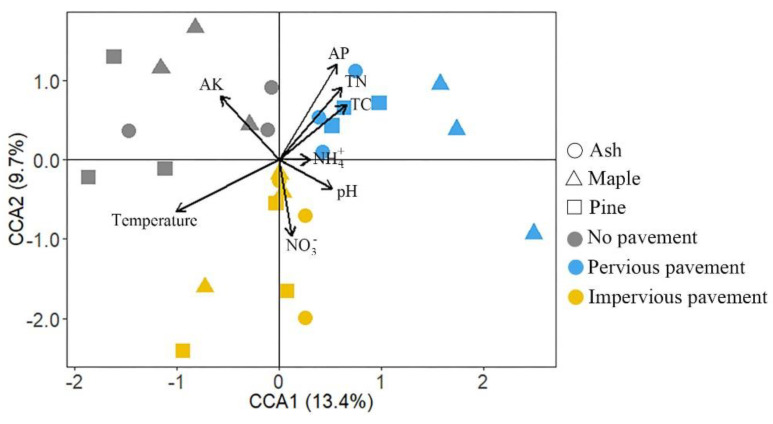
Canonical correlation analysis (CCA) ordination diagrams of soil bacterial communities and soil variables defined by the first and second axes.

The results of canonical correlation analysis (CCA) revealed a close correlation between soil bacterial community composition and environmental factors, in which the first and second CCA axis explained 13.4% and 9.7% of the variance, respectively (Figure 4). Soil temperature, AP, AK, and TN played the primary roles in the shift in bacterial community composition, as they were represented by longer arrows when compared with the other soil variables (i.e., pH, NO_3_^−^, NH_4_^+^, and TC). Mantel tests corroborated this finding, indicating a strong correlation between soil temperature and bacterial community composition (Table 2). Further, the bacterial community exhibited significant correlations with soil temperature, AP, TC, and TN based on the Mantel test (Table 2). The results of a linear mixed-effects model demonstrated that factors that influenced soil bacterial Shannon diversity included pH and NH_4_^+^ (Table 3). In addition, soil bacterial richness was correlated negatively and remarkably with soil temperature and TC, but positively with soil pH and TN (Table 3). 

## 4. Discussion

Elucidation of the effects of anthropogenic disturbances on soil microbial communities is conducive to our understanding of microbial adaptation to the variations in biotic and abiotic factors both above- and belowground [25]. In this study, impervious pavement changed soil bacterial community composition and reduced bacterial diversity. These results supported our first hypothesis that pavement alters soil bacterial composition and decreases their diversity. Contrary to our expectation, soil bacterial communities did not vary with tree species, and thus, we rejected our second hypothesis. There were no significant differences in soil bacterial diversity between pervious pavement and no pavement, which supported our third hypothesis. 

Anthropogenic disturbance, such as the conversion of land cover, has a significant effect on bacterial communities by changing their edaphic properties [26,27]. Our results demonstrated a different bacterial community composition in pavement soil compared to no pavement soil, which is consistent with previous reports [13,24]. With respect to the bacterial phyla, *Actinobacteria*, *Chloroflexi*, *Proteobacteria*, *Acidobacteria*, *Firmicutes*, and *Gemmatimonadetes* were found to be the dominant phyla, consistent with previous studies that have investigated disturbed soils in urban areas [28,29]. The relative abundances of the primary phyla, *Proteobacteria*, *Chloroflexi*, and *Actinobacteria*, in pavement soil were similar to that in no pavement soil, which is inconsistent with a previous study [13]. This may be explained by the minor changes in soil pH, carbon, and nitrogen under impervious pavement in our study. In contrast, *Firmicutes*, which are resistant to extreme environmental changes, had a greater relative abundance in pavement soil than no pavement soil. Hu et al. (2018) observed that the difference in soil bacterial community structure between impervious and pervious pavement was small compared with open areas [13]. However, in this study, the samples beneath pervious pavement differed distinctly from those beneath no pavement and impervious pavement based on NMDS.

Soil bacterial diversity was related to material exchange and energy flow between aboveground and belowground. Previous studies have found that soil microbial biomass and bacterial diversity decreased beneath impervious surfaces because the soil nutrients were depleted. As hypothesized, soil bacterial diversity and richness, measured by the Shannon and Chao 1 indices, decreased with the installment of impervious pavement. However, pervious pavement that has been proven better than impervious pavements could potentially provide an improved environment for soil, as well as increase microbial biomass, enzyme activity, and functional diversity [11,12]. In this study, soil bacterial diversity was similar between pervious pavement and no pavement. These results suggested that, in terms of biodiversity, pervious pavement can mitigate the negative effects of impervious pavements.

The relation between belowground microorganisms and plants is often said to be complex and have direct or indirect effects on microbial communities. Many studies have proven that tree species shape the structure and function of soil microbial communities because of root exudates [30,31]. Moreover, soil microorganisms use leaf litter as a substrate in forest ecosystems, which is one of the main determinants of soil microbial structure and diversity that vary with plant species [32,33]. In addition, metabolic rates of soil bacteria were lower in conifer forest ecosystems than in deciduous forest ecosystems [34]. However, abiotic factors, such as land-use history, affected soil microbial communities far more than they did aboveground plants [35]. In this study, pavement had a greater effect on soil bacterial community structure and diversity than did tree species five years after planting. This may be because pavement impeded the inputs of leaf litter, which impaired the vegetation’s effects on soil microbial communities. Moreover, pavement’s effects on soil microbial communities varied with plant species. In our previous study, soil fungi, arbuscular mycorrhizal fungi, and the fungi: bacteria ratio were reduced significantly under both impervious and pervious pavements in pine stands, while for ash stands, they were decreased significantly only under pervious pavements. We observed that pavement had a greater effect on soil bacterial community composition in pine stands, followed by ash and maple stands. In addition, impervious pavement decreased both soil bacterial diversity and richness indices in pine stands, which was not observed in ash and maple stands. These results indicated that soil bacterial communities in pine stands were more sensitive to impervious pavement than were those in ash and maple stands.

Soil microbial community composition and diversity are associated closely with soil habitat properties. A previous study revealed that impervious surfaces induced changes in soil physicochemical properties, including temperature, pH, TC, TN, SOC, NO_3_^−^-N, and AP [36]. Soil pH increased, but TC, SOC, and TN were depleted under impervious surfaces, which stems from the need to remove carbon and nitrogen-rich topsoil before sealing [10]. Lu et al. reported that over 90% of soil total carbon and total nitrogen were lost under impervious surfaces [37]. Interestingly, only 13% and 19% of soil TC and TN, respectively, were lost beneath impervious pavement in this study, which may be because the topsoil was not removed in our field experiment. However, the variation in soil NO_3_^−^-N and AP with pavement was similar to that in a previous study [13]. Many studies have found that soil bacterial communities are sensitive to temperature shifts. Here, soil temperature, which was 2 °C higher beneath impervious pavements than no pavements, was the most important determinant of variation in soil bacterial community composition. Moreover, Dennis et al. (2019) observed that soil bacterial diversity was related positively to atmospheric temperature [38]. Although there was no significant difference in surface temperature among pavements in the study area, soil temperature increased under impervious pavement and was correlated negatively with soil bacterial diversity. It has been shown that soil bacterial community composition, microbial biomass, and functional diversity are altered by changes in nutrient availability, such as SOM, NO_3_^−^-N, NH_4_^+^-N, AP, and AK [11,13], which our results corroborated. Further, a strong correlation between soil bacterial community diversity and pH and nutrients, including TC, TN, and NO_3_^−^-N was observed in this study.

## 5. Conclusions

To conclude, our results showed that pavement was a more important predictor of soil bacterial community composition and diversity than tree species via changing soil properties. In addition, the effects of pavement on soil bacterial communities varied with tree species and were stronger in pine stands than in ash and maple stands. As expected, with respect to bacterial diversity, pervious pavement was a good choice to alleviate the adverse effects of impervious surfaces on microorganisms. Soil temperature, pH, and nutrients were also found to be the critical factors that shape soil bacterial communities. However, the duration of this field experiment was five years, and more long-term work should be considered to obtain a full understanding of the response of microorganisms to human disturbance. These novel results highlight the importance of pavement when exploring the role of anthropogenic interference in determining the community composition and diversity of soil bacteria underground.

## Figures and Tables

**Table 1:** Bacterial diversity and richness indices calculated from 16S rRNA gene sequence data at OTU level. Different letters indicate a significant difference among pavements at *p* < 0.05 based on Tukey’s HSD test.

Pavement	Pine		Ash		Maple	
	Chao 1	Shannon	Chao 1	Shannon	Chao 1	Shannon
IPP	2063 ± 149 b	10.11 ± 0.05 b	1987 ± 337 b	9.97 ± 0.05 a	2310 ± 332 a	10.09 ± 0.07 a
PP	2507 ± 199 a	10.24 ± 0.04 a	2133 ± 271 b	10.04 ± 0.04 a	2196 ± 361 a	10.24 ± 0.02 a
NP	2672 ± 86 a	10.26 ± 0.01 a	3052 ± 132 a	10.26 ± 0.11 a	2822 ± 208 a	10.26 ± 0.09 a

IPP: Impervious pavement, PP: Pervious pavement, NP: No pavement.

**Table 2:** Statistical results of Mantel tests evaluating the relation between soil environmental variables and bacterial community structure. Significant effects (*p* < 0.05) are shown in bold.

Variable	R	*p*
Temperature	0.397	0.001
AP	0.286	**0.004**
TC	0.233	**0.006**
TN	0.198	**0.037**
PH	0.097	0.146
NH_4_^+^	0.101	0.824
NO_3_^-^	0.040	0.525
AK	0.030	0.407

**Table 3:** Effects of soil variables on bacterial richness (Chao 1) and diversity (Shannon) explored with a linear mixed-effects model. *p*-values from likelihood ratio tests (df = 25). Significant effects (*p* < 0.05) are shown in bold.

Index	Fixed Effects	Estimate	Std Error	*t*-Value	*p*-Value
Chao 1	(Intercept)	0	0.128	0	1.000
	temperature	−0.387	0.173	−2.236	**0.041**
	PH	0.411	0.155	2.646	**0.013**
	TC	−1.302	0.437	−2.979	**0.006**
	TN	1.364	0.502	2.718	**0.012**
	NH_4_^+^	0.031	0.127	0.246	0.808
	NO_3_^−^	−0.351	0.181	−1.945	0.063
	AP	−0.014	0.272	−0.050	0.960
Shannon	(Intercept)	0	0.151	0	1.000
	temperature	−0.143	0.201	−0.711	0.488
	PH	0.414	0.177	2.343	**0.027**
	NO_3_^−^	−0.969	0.495	−1.957	0.062
	TC	1.039	0.568	1.829	0.080
	TN	−0.030	0.144	−0.211	0.834
	NH_4_^+^	−0.449	0.205	−2.19	**0.038**
	AP	0.084	0.309	0.272	0.788

## Supplementary Materials

The following are available online at https://www.mdpi.com/1660-4601/18/4/2168/s1, Figure S1: Layout of the experiment field. Figure S2: Soil physical and chemical properties under different pavements in pine, ash and maple stands in study area. One-way ANOVA (*n* = 27, df = 2) was performed to test the effects of pavement on soil properties. Different letters mean significant difference at *p* < 0.05 among the land pavements. TC: total carbon; TN: total nitrogen; AP: available phosphorus; AK: available potassium. Figure S3: Heatmap showing the relative abundance of the top 50 genera for bacterial community. The samples were clustered according to Euclidean distances. The colors correspond to the relative abundance of each genus in the samples (indicated by the color legend). S1 Supplemental Methods.
