# Supplementary material for: Pavement Overrides the Effects of Tree Species on Soil Bacterial Communities
Source: Int J Environ Res Public Health. 2021 Feb 23;18(4):2168. doi: 10.3390/ijerph18042168 (PMC7927126; doi:10.3390/ijerph18042168)
Supplement: Supplementary file 1 [file ijerph-18-02168-s001.pdf]

Supplementary data

Table S1. Soil mean, lowest and highest (in parentheses) temperature from January to October 2017 under different land pavements in pine, ash and maple stands in study area. One-way ANOVA (n = 9, df = 2) and Tukey's HSD test were performed and significant differences between pavements or tree species are indicated by different letters ($P < 0.05$).

Pavement	Soil temperature (°C)	Plant species	Soil temperature (°C)
Impervious pavement	15.34 (-3.03 to 29.57)a	Pine	15.24 (-2.73 to 29.57)
Pervious pavement	15.05 (-2.73 to 26.82)a	Ash	14.51 (-3.03 to 26.08)
No pavement	13.21 (-1.73 to 25.08)b	Maple	13.91 (-0.36 to 25.83)
P	0.039		0.482

Figure S1. Layout of the experiment field.

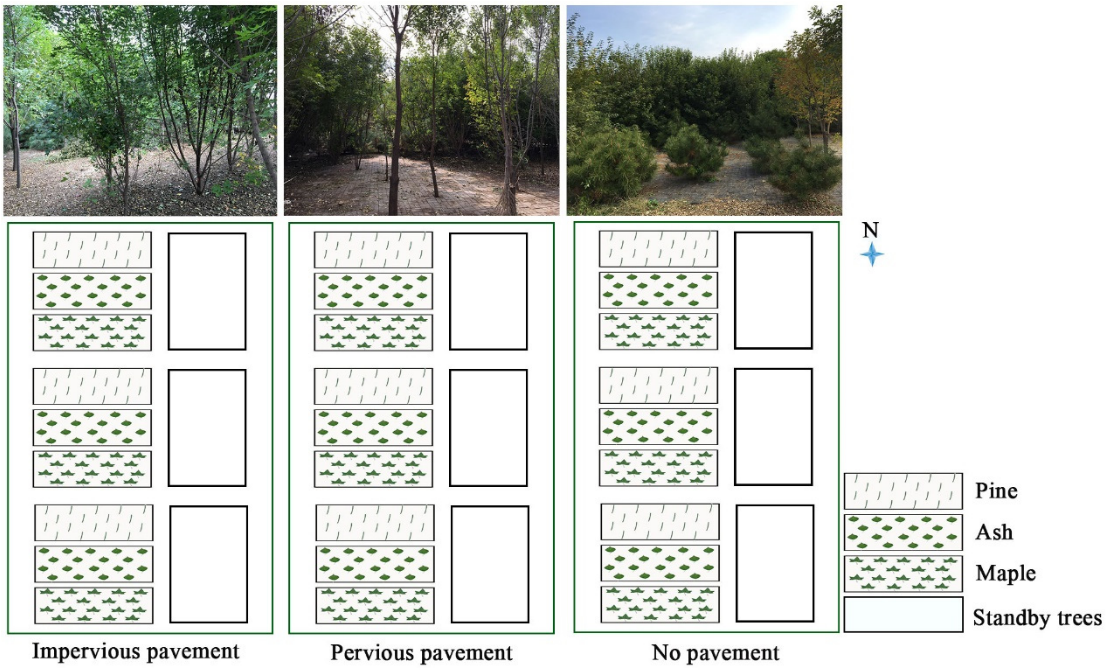

Figure S2. Soil physical and chemical properties under different pavements in pine, ash and maple stands in study area. One-way ANOVA ($n = 27$, $df = 2$) was performed to test the effects of pavement on soil properties. Different letters mean significant difference at $P < 0.05$ among the land pavements. TC: total carbon; TN: total nitrogen; AP: available phosphorus; AK: available potassium.

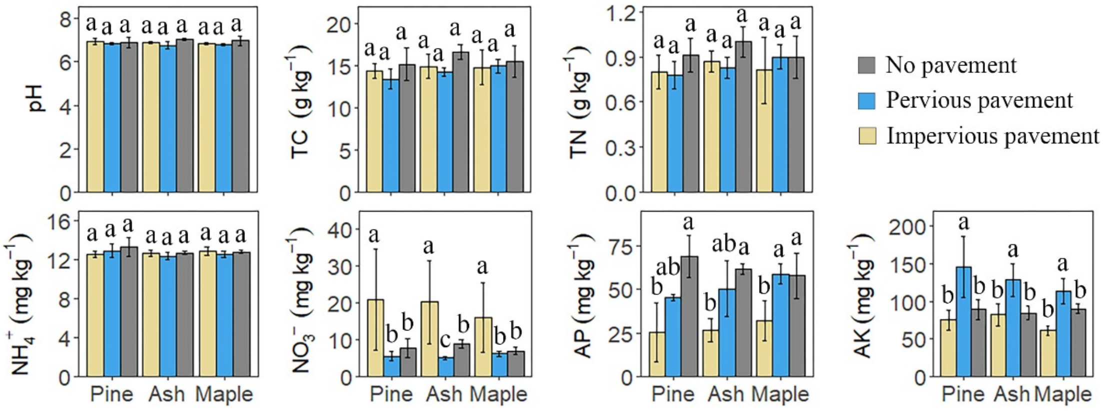

Figure S3. Heatmap showing the relative abundance of the top 50 genera for bacterial community. The samples were clustered according to Euclidean distances. The colors correspond to the relative abundance of each genus in the samples (indicated by the color legend).

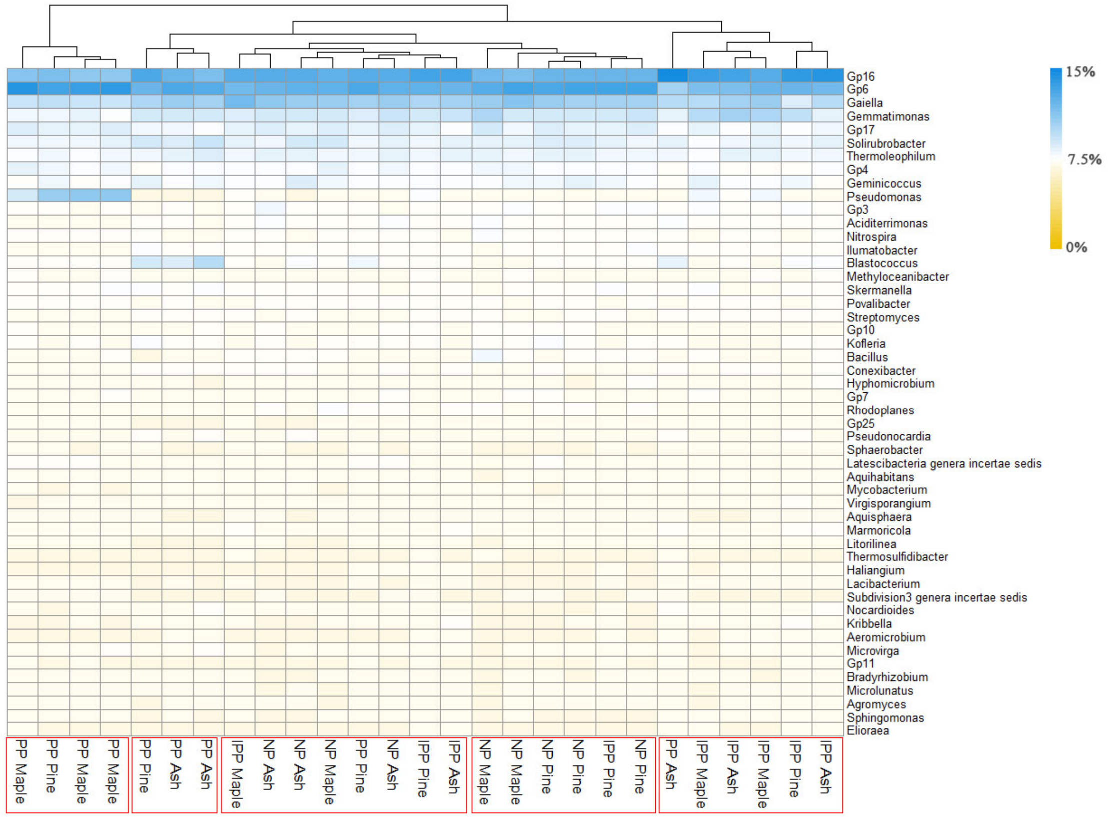

S1 Supplemental Methods

PCR amplification and 16S rDNA sequencing

The V3-V4 regions of the bacterial 16S RNA gene were amplified using primers 338F (5′-ACTCCTACGGGAGGCAGCA-3′) and 806R (5′- GGACTACHVGGGTWTCTAAT-3′). The amplification conditions were: 98 °C for 5 min; 25 cycles of 98 °C for 30 s, 50 °C for 30 s, and 72 °C for 30 s; and a final extension at 72 °C for 5 min. PCR amplicons were extracted from 2% agarose gels and purified using an AxyPrep DNA Gel Extraction Kit (Axygen Biosciences, Union City, CA, USA) according to the manufacturer's instructions. The purified amplicons were equimolarly mixed for library construction using the TruSeq DNA kit, and 2 × 300 bp paired-end sequencing was carried out on an Illumina MiSeq sequencer (Illumina Inc., San Diego, USA).

Bioinformatics analysis

Reads with low quality (<20 score) or ambiguous bases were removed. Reads did not match the primer and barcode were also discarded. UPARSE algorithm was used to remove chimeras and generate OTU (operational taxonomic units) table at a 97% similarity level. The representative sequence of each OTU was selected and classified using the platform Ribosomal Database Project (RDP).
